# Adsorption of sulfur into an alkynyl-based covalent organic framework for mercury removal†

**DOI:** 10.1039/d2ra06838a

**Published:** 2022-12-12

**Authors:** Shenglin Wang, Yingxiang Xin, Hui Hu, Xiaofang Su, Jifeng Wu, Qianqian Yan, Jiaying Qian, Songtao Xiao, Yanan Gao

**Affiliations:** Key Laboratory of Ministry of Education for Advanced Materials in Tropical Island Resources, Hainan University No 58, Renmin Avenue Haikou 570228 China ygao@hainanu.edu.cn sxf@hainanu.edu.cn; Dalian Institute of Chemical Physics, Chinese Academy of Sciences 457 Zhongshan Road Dalian 116023 P. R. China; China Institute of Atomic Energy Beijing 102413 P. R. China xiao200112@163.com

## Abstract

Highly efficient removal of Hg(ii) has been previously achieved through the adsorption by functionalized covalent organic frameworks (COFs). Among these COFs, thioether groups need to be deliberately introduced into the pores of COFs through either a bottom-up synthesis or post-synthesis strategy. Herein, we report a simple mercury removal strategy that used a stable alkynyl (–C≡C–) based covalent organic framework (TP-EDDA COF) as an adsorbent for Hg(ii) removal. Sulfur vapor was first adsorbed by the TP-EDDA COF due to the van der Waals interaction between adsorbed sulfur and alkynyl groups. The Hg(ii) removal capability was then evaluated for the sulfur loaded TP-EDDA COF. Our results exhibited a good Hg(ii) removal performance for the sulfur loaded TP-EDDA COF. It was deduced that s⋯π interaction between sulfur atom and the alkynyl groups of the COF skeleton caused an increase in the electron density of sulfur and the electronegative sulfur atoms acted as a soft acid to accept soft-basic Hg(ii). This strategy provides a convenient platform for COFs to cope with environmental issues.

## Abstract 2

A simple mercury removal strategy was developed that used a stable alkyl based covalent organic framework to adsorb sulfur first and then served as an adsorbent to remove Hg(ii) effectively.

## Graphical abstract

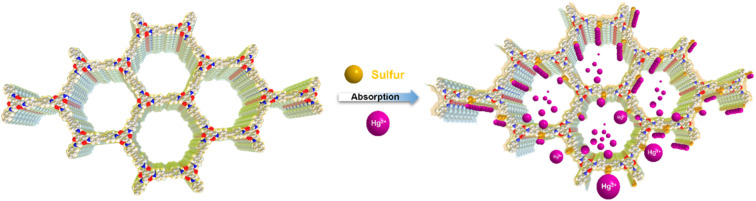

## 1 Introduction

Mercury has long been considered as the most hazardous water pollutant that causes several health and environmental problems even at extremely low concentrations.^1^ To date, various treatment strategies have been explored for the removal of mercury from the environment, including precipitation,^2^ membrane separation,^3^ ion-exchange,^4^ physical and chemical adsorption,^5^ and so on. As attractive candidates for mercury capture, porous materials are of particular interest because of their intrinsic porosity giving a high mercury adsorption capacity. Activated carbon,^6^ zeolites,^7^ mesoporous and microporous silica,^8^ and metal–organic frameworks (MOFs)^9^ have been already applied for Hg(ii) removal. However, developing novel adsorbent materials to remove mercury completely and efficiently is still highly desirable nowadays.

Covalent organic frameworks (COFs) are an interesting class of porous organic microcrystalline polymers constructed by reversible chemical bonds, and feature high specific surface area, low density, light-weight elements, and designed structures.^10,11^ These materials have attracted great attention from scientists in many fields such as catalysis,^12–15^ semiconductors,^16–18^ optical sensors,^19–21^ energy storage,^22–24^ and mass transport.^25–27^ COFs enable the predesigned porous structures through the topology diagram and post-synthesis modification, which provides a promising porous platform for the capture of various target gas molecules, like hydrogen,^28,29^ carbon dioxide,^29–31^ methane,^29,32^ and ammonia.^33,34^ Due to structural designability and functional diversity of COFs, some attempts have also been made to develop COFs for mercury removal. Wang and his co-workers first reported a thioether functionalized COF-LZU8 that was synthesized based on a long-chain dialkylthioether building unit through a bottom-up synthesis strategy and the COF demonstrated highly sensitive detection and effective removal of Hg^2+^ from acetonitrile.^35^ In a similar way, a short sulfide functional (methyl sulfide) building unit was used to construct TPB-DMTTPA-COF to effectively remove Hg(ii) from aqueous solutions by Jiang *et al.*^36^ Additionally, post-synthesis modification method was also used to construct thioether-based COFs. With this strategy, vinyl and ethynyl building units were integrated into the skeleton of maternal COFs and thiol functional groups were then grafted within the pores of the COFs through chemical reactions.^37,38^ Among these thioether COFs, TPB-DMTPCOF-SH demonstrated the highest recorded Hg(ii) uptake capacity (4395 mg g^−1^ in water) with an exceptional high distribution coefficient value (*K*_d_ of 3.23 × 10^9^).^38^ Although these COFs exhibited outstanding mercury removal capacities, it is not easy to design and prepare functional COFs in a desirable fashion, especially for COFs that are constructed by bottom-up strategy since the long thioether groups would hinder the crystallization of COFs. In contract to bottom-up strategy, post-synthetic integration of chemical active sites into the skeleton of COFs is considered to be a powerful strategy to obtain functional COFs, with which thioether groups can be grafted onto the COF skeleton through the reactions with active sites. However, the harsh solvothermal conditions would lead to the undesired effect on the catalytic sites and post-synthetic process would also cause a decreased crystallization of COFs. Therefore, it will be of great significance to develop COFs that can remove Hg(ii) simply and effectively.

It is known that sulfur is the privileged receptor for Hg according to the soft and hard acid base theory.^35^ COFs have been widely used as the host for sulfur in the field of lithium–sulfur (Li–S) batteries.^39–41^ Large quantities of sulfur can be absorbed within the pores of COFs by thermal evaporation in sulfur saturated vapor. Based on these facts, we here propose a simple strategy to remove Hg(ii) by COFs. A stable alkynyl (–C≡C–) based COF, TP-EDDA COF, was first prepared as a host and sulfur vapor was then loaded within the pores of the COF. The resulting sulfur-loaded COF, S@TP-EDDA COF, was used as an adsorbent for Hg(ii) remove from ethanol and exhibited good Hg(ii) removal capability. This strategy presents a simple and effective way to remove Hg(ii), opening up a new way for COFs to cope with world-threatening pollution issues.

## 2 Experimental section

### 2.1. Materials

All the reagents and solvents used in this work were obtained from commercial sources and used without further purification unless otherwise specified. Two building units, 1,3,5-triformylphloroglucinol (TP) and 4,4′-(ethyne-1,2-diyl)dianiline (EDDA) were synthesized according to the reported procedures.^42,43^ The ^1^H NMR spectra of both EDDA and TP matched well with those reported in literature (see ESI section, Fig. S1 and S2†).

### 2.2. Synthesis of TP-EDDA COF

**Scheme 1:**
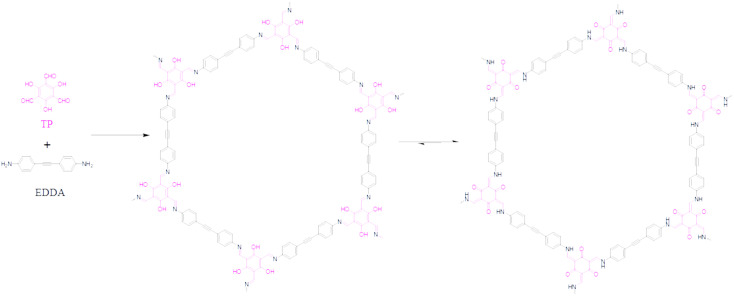
Synthesis of TP-EDDA COF.

TP-EDDA COF was synthesized according to a modified method (Scheme 1).^44^ In a typical procedure, TP (12.6 mg, 0.06 mmol) and EDDA (19.8 mg, 0.09 mmol) were loaded in a glass ampule vessel (10 mL). Then, a 1.0 mL mixed solution of mesitylene/1,4-dioxane (1/1 by volume) was added to the vessel. After the mixture was sonicated for 10 min, 0.1 mL acetic acid (6.0 M) was rapidly added. The vessel was sonicated for another 5 min and then flash-frozen in liquid nitrogen. The reaction system was degassed through freeze–pump–thaw cycles for three times. The system was sealed (<4 Pa) with a flame, and then heated at 120 °C for 3 days. A precipitate was obtained after filtration and washed thoroughly with *N*,*N*-dimethylformamide (DMF) and tetrahydrofuran (THF), successively, to produce an orange powder. The powder was dried at 120 °C under vacuum overnight to give the product in 74% yield.

### 2.3. Synthesis of S@TP-EDDA COF

Sulfur was loaded within the TP-EDDA COF with a traditional method according to a previous report.^45^ Typically, a vial containing 10.0 mg of TP-EDDA COF and an excessive dose of commercial sulfur (*ca.* 0.1 g) were put in a closed system. After that, the closed system was heated at 155 °C for a desired time under Ar atmosphere. The obtained product was named as S@TP-EDDA COF.

### 2.4. General procedure for Hg(ii) removal

To a 25 mL single-neck round bottle flask was added 5 mg COF sample and 5 mL HgCl_2_ ethanol solution (10 mg L^−1^). The pH of the solution was adjusted to a desired value and the suspension was stirred at 25 °C for 3 hours. The Hg(ii) concentration was determined by inductively coupled plasma mass spectrometry (ICP-MS).

### 2.5. Methods

Power X-ray diffraction (PXRD) characterizations were recorded on a PANalytical X'Pert model Pro Multipurpose Diffractometer that uses Cu Kα radiation at 40 kV and 40 mA. The data were collected in the range of 2.5–30° (2*θ*) at 0.03° step scan with exposure time of 10 s per step. Fourier transform infrared (FT-IR) spectra were measured by a Bruker model TENSOR 27 spectrophotometer (KBr pellets). Nitrogen adsorption–desorption measurements were collected by using a Quantachrome AutosorbiQ2 analyzer using adsorbates of UHP grade. The freshly-prepared samples were first activated at 100 °C for 15 h under vacuum before the measurement. The specific surface areas were evaluated by the Brunauer–Elmett–Teller (BET) model with desorption branches over *P*/*P*_0_ of 0.01–0.05. The pore size distributions of COFs were evaluated by the nonlocal density functional theory (NLDFT) method. Thermogravimetric analyses (TGA) were performed by a STA449F3 analyzer, Netzsch, Germany, under nitrogen atmosphere (heating rate of 10 °C min^−1^; N_2_ flow rate of 20 mL min^−1^) from room temperature to 1000 °C. ^1^H NMR spectra were recorded by a Bruker Advance III 400 MHz NMR spectrometer (Bruker BioSpin Corporation, Fällanden, Switzerland). Scanning electron microscopy (SEM) images were taken on a FEI Quanta 200F operating at an accelerating voltage of 20 kV. The metal content was analyzed by a PerkinElmer Elan DRC II quadrupole inductively coupled plasma mass spectrometer (ICP-MS) analyzer.

## 3 Results and discussion

### 3.1 Characterization of TP-EDDA COF

**Fig. 1:**
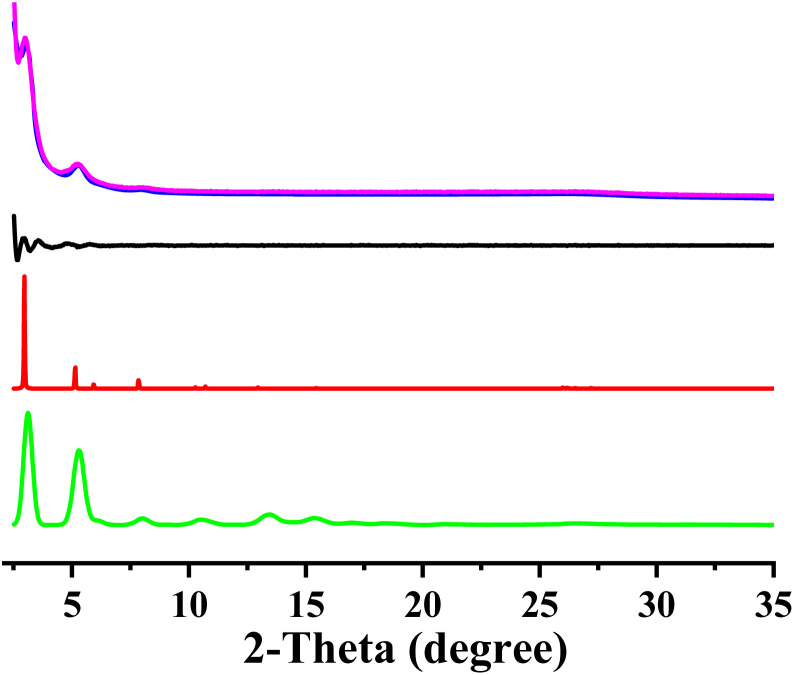
Observed XRD pattern (magenta); profile simulated using the Pawley refinement (bule); their difference (black); AA-stacking mode (red) and AB-stacking mode of the TP-EDDA COF.

The optimum reaction condition for the formation of crystalline COF was first screened (Fig. S3†). TP-EDDA COF can be obtained under several different reaction conditions. The optimum solvent combination was mesitylene/1,4-dioxane in 1/1 by volume. The successful synthesis of TP-EDDA COF can be confirmed by powder X-ray diffraction (PXRD) and Fourier transform infrared (FT-IR) spectroscopy. The crystallization and unit cell parameter of TP-EDDA COF were first analyzed by PXRD together with computational simulations. Several diffraction peaks of the TP-EDDA COF appeared at 3.1°, 5.3°, 6.1°, and 8.0°, which can be attributed to the (100), (110), (200), and (210) facets, respectively (Fig. 1, magenta curve). A broad reflection at 26.9°, which was assigned to the (100) facet. This reveals the π–π stacked structure of a 2D COF. The structural model of TP-EDDA COF was built with the expected 2D stacked hexagonal pores. The use of lattice modeling and Pawley refinements produce an eclipsed AA stacking arrangement that can reproduce the experimentally measured PXRD pattern well (Fig. 1, blue curve, red curve) with negligible difference (Fig. 1, black curve). A unit cell of *P*6/*m* with the parameters of *a* = *b* = 34.40 Å, *c* = 3.47 Å, *α* = *β* = 90° and *γ* = 120° were deduced, which is basically in accordance with the previously reported result.^44^ The *R*_wp_ and *R*_p_ values were converged to 4.50% and 3.44%, respectively. In contrary, the diffraction patterns of the staggered stacking models did not reproduce the data (Fig. 1, green curve). The morphology of TP-EDDA COF was characterized by SEM (Fig. S4†). It was shown that a large amount of granular particles with an average size of about dozens of nanometers were observed, which is different from the reported result where a flower-like morphology with dimensions in the 100 nm range was obtained.^44^ This suggests that different synthesis condition afforded COF crystallites with different morphology.

**Fig. 2:**
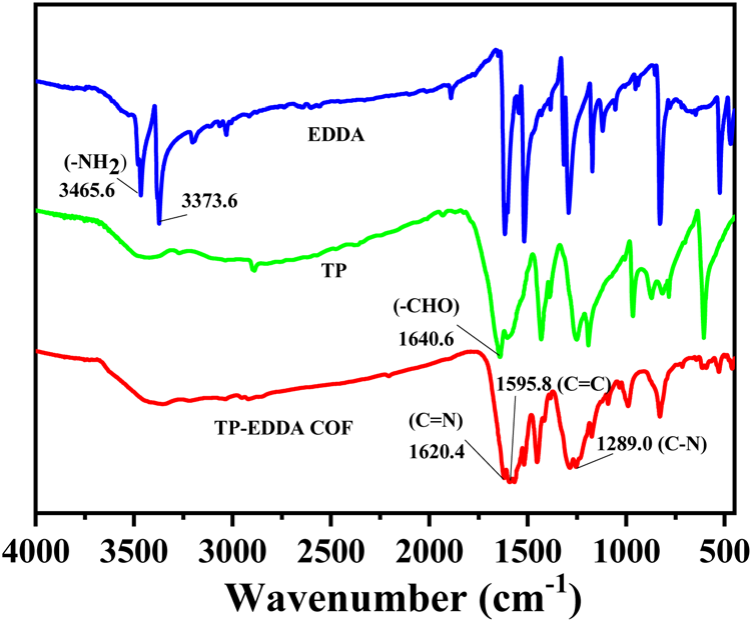
FT-IR spectra of TA, EDDA and TA-EDDACOF.

**Fig. 3:**
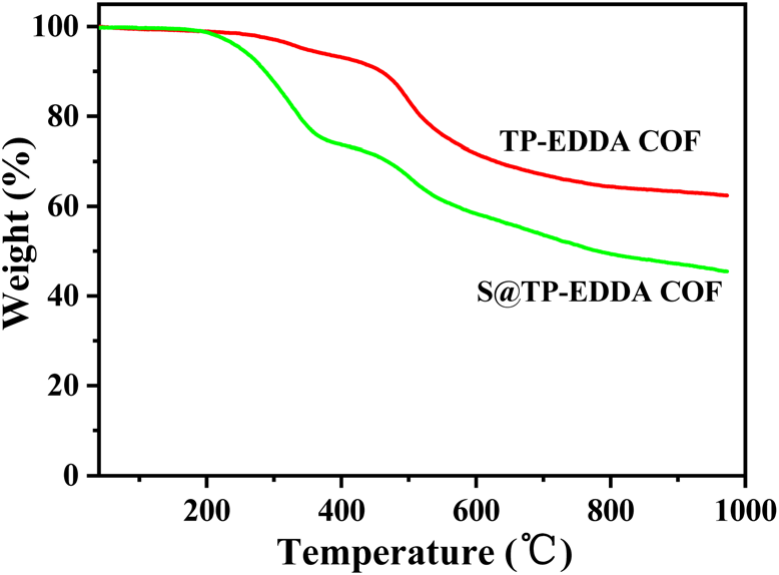
TGA curves of TP-EDDA COF and S@TP-EDDA COF.

FT-IR spectra also confirm the formation of TP-EDDA COF (Fig. 2). The absence of aldehyde stretching band (1640.6 cm^−1^) of TP and characteristic peaks of amino stretching (3465.6 cm^−1^ and 3373.6 cm^−1^) of EDDA shows the total consumption of reactants. Several new strong peaks were observed in TP-EDDA COF. Among them, the peak at 1450 cm^−1^ was due to carbonyl group and the peaks at 1289 and 1090 cm^−1^ can be attributed to the stretching vibrations of aryl secondary amine and C–N, respectively, which indicates that the skeleton of the COF underwent an enol–keto transfer.^45^ Besides, a strong peak was also observed at 1175 cm^−1^ that can be derived from phenolic hydroxyl group and the peak at 1620.4 cm^−1^ was ascribed to the imine group. This result means that the enol–keto tautomerism is not fully complete. TGA of TP-EDDA COF was performed to determine the thermal stability. It is clear that the decomposition temperature of the COF reached up to 490 °C (Fig. 3, red curve). A slight decrease at about 300 °C may be due to the adsorbed guest molecules and impurities. The chemical stability of TP-EDDA COF was further carried out. The COF samples were dispersed in different solvents, including methanol, hexane, water and aqueous HCl (1 M) and NaOH (1 M) solutions, at 25 °C for 3 days. All samples (except in 1 M HCl) exhibited intense PXRD patterns without obvious change either in the peak position or the intensity, indicating that the high crystallinity is retained under these harsh conditions (Fig. S5†). This result suggests a good chemical stability of the TP-EDDA COF.

**Fig. 4:**
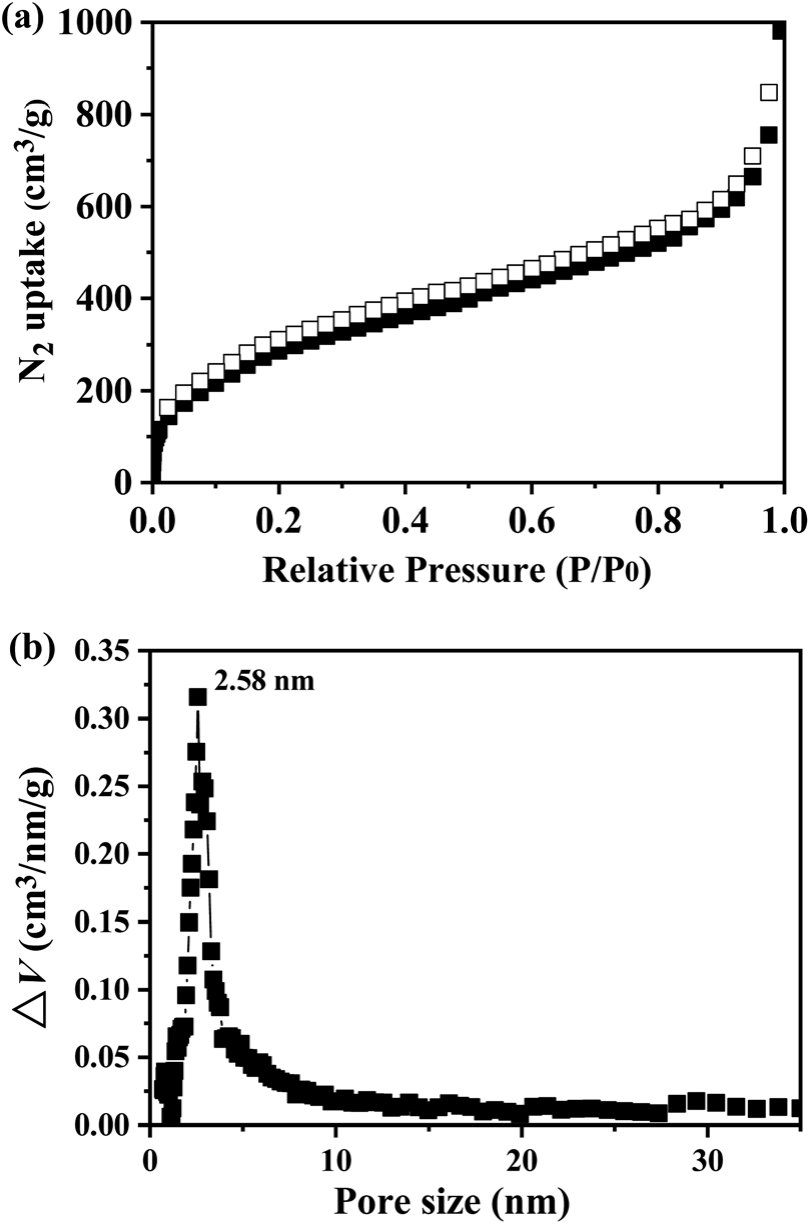
Nitrogen adsorption and desorption isotherm of TP-EDDA COF measured at 77 K (a) and pore size distribution (b) by fitting NLDFT to the adsorption data.

The porosity of TP-EDDA COF was further estimated by nitrogen adsorption–desorption isotherm recorded at 77 K (Fig. 4a). The BET surface area of the TP-EDDA COF was calculated to be 1183 m^2^ g^−1^ and the pore volume was estimated to be 1.83 m^3^ g^−1^ (*P*/*P*_0_ = 0.99). The pore size of the TP-EDDA COF was estimated to be 2.58 nm according to the nonlocal density functional theory (NLDFT) calculation (Fig. 4b). The size is close to the theoretical value estimated by PM3 simulations.

### 3.2 Adsorption of sulfur by TP-EDDA COF

**Fig. 5:**
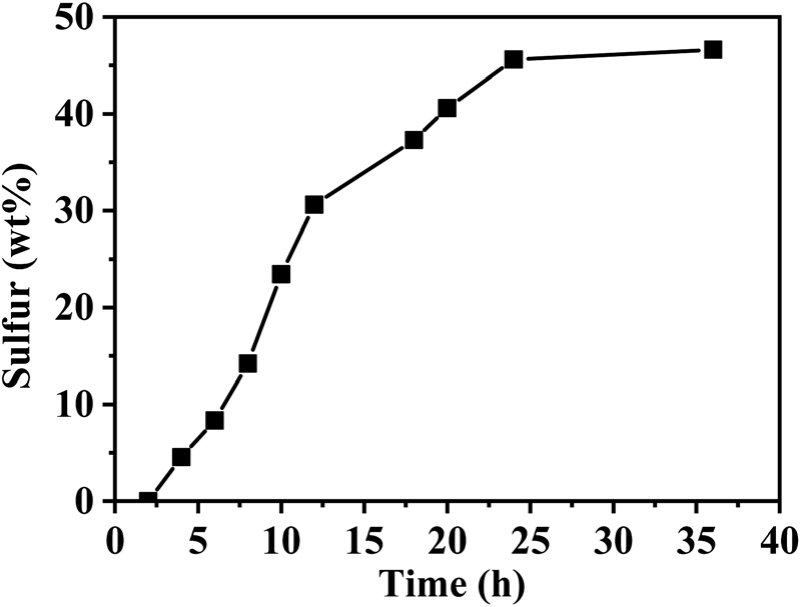
Gravimetric surfer uptake of TP-EDDA COF as a function of time at 155 °C.

The adsorption behavior of sulfur vapour by the TP-EDDA COF was shown in Fig. 5. The sulfur adsorption amount increased with adsorption time and reached saturation when adsorption time was 25 hours. We chose S@TP-EDDA COF at 12 hours as a model for the following investigation. At this time, the sulfur content was about 28.6 wt%. The PXRD of S@TP-EDDA COF was also first detested (Fig. S6†). It can be seen that the strong diffraction peak at 3.1° disappeared, which indicated that the pores of the COF was filled with sulfur atoms.^46^ Also, the characteristic diffraction peaks of sulfur disappeared, revealing that sulfur atoms were highly dispersed within the pores of the TP-EDDA COF. The nitrogen adsorption–desorption isotherm of S@TP-EDDA COF also confirmed the adsorption of sulfur into the pores of the COF (Fig. 4). The BET surface area was remarkable decreased from 1183 to 123 m^2^ g^−1^ and the pore volume decreased from 1.83 to 0.37 m^3^ g^−1^ when sulfur was loaded. Additionally, TGA showed that a weight loss of S@TP-EDDA COF happened at 200 °C (Fig. 3, green curve), which is lower than the decomposition temperature of TP-EDDA COF, suggesting the loading of sulfur into the COF.

**Fig. 6:**
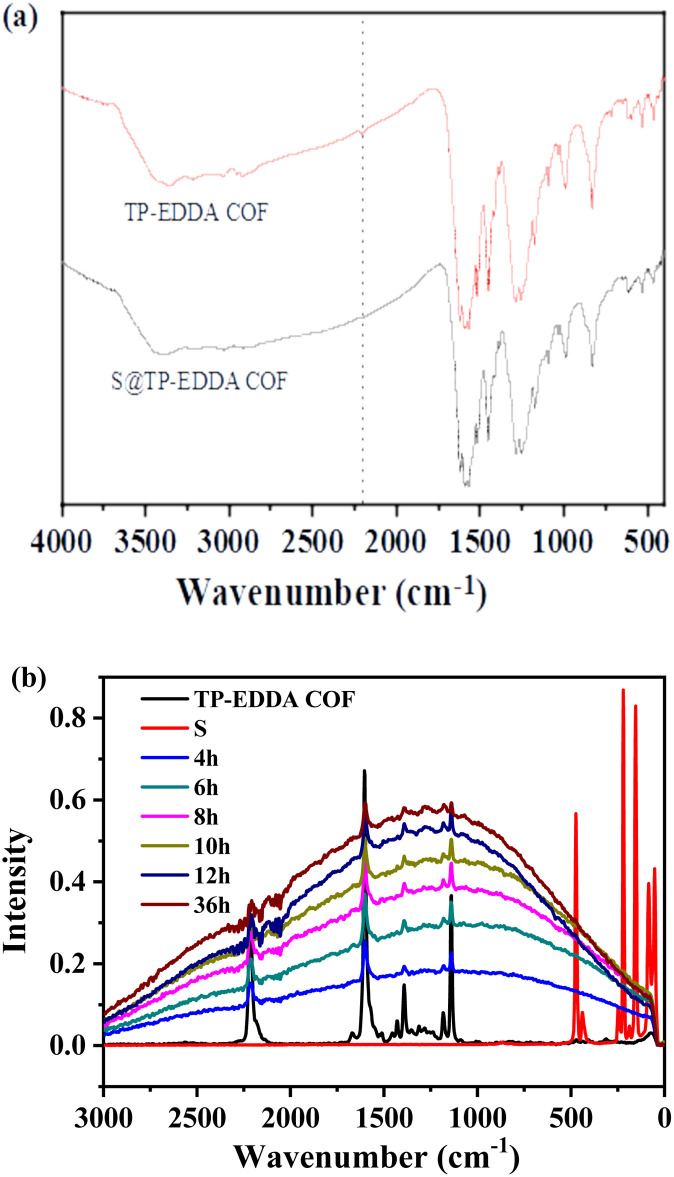
FT-IR spectra of TP-EDDA COF and S@TP-EDDA COF (a) and Raman spectra of TP-EDDA COF and sulfur loaded TP-EDDA COF at different times (b).

To probe into the interaction between the sulfur and the TP-EDDA COF, FT-IR and Raman spectra were used to analyse the adsorption mechanism. After loading sulfur vapour, the characteristic peak of alkynyl group at 2206 cm^−1^ disappeared, suggesting the strong interaction between the sulfur and the skeleton of TP-EDDA COF. Meanwhile, no new peak due to –C–S– bonds appeared at *ca.* 980 cm^−1^ in the S@TP-EDDA COF spectrum (Fig. 6a). This suggests that no chemical reactions happened between sulfur and the skeleton of the TP-EDDA COF. Raman spectra showed that the intensity of the peak of –C≡C– at 2200 cm^−1^ became weak gradually with adsorption time (Fig. 6b), also revealing the existing of strong interaction between sulfur and the COF skeleton. We believe that π-electron-rich alkynyl groups may transfer electrons to sulfur atoms and formed the charge transfer complexes.^47^

### 3.3. Hg(ii) removal by TP-EDDA COF

**Fig. 7:**
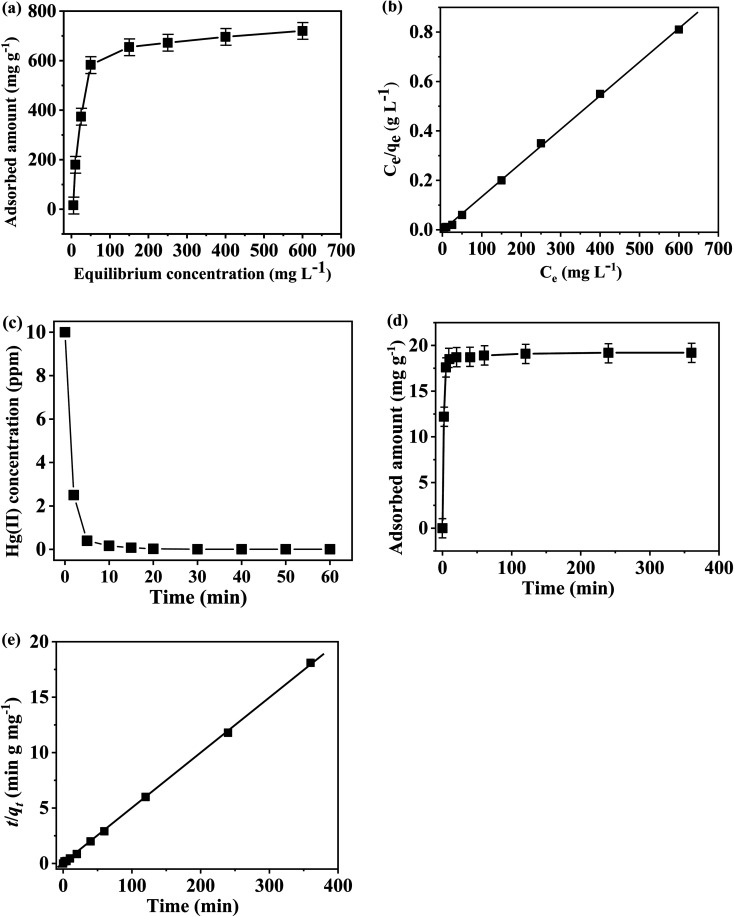
(a) Hg(ii) adsorption isotherm (24 h). (b) Linear regression by fitting the equilibrium adsorption data with Langmuir adsorption model. (c) Hg(ii) sorption kinetics at the initial Hg(ii) concentration of 10 ppm. (d) Adsorption curve of Hg(ii) at different contact time in ethanol solution. (e) Pseudo-second-order kinetic plot for the adsorption at Hg(ii) concentration of 10 ppm.

The Hg(ii) removal capability of the TP-EDDA COF was detected by ICP-MS. We found that the equilibrium adsorption isotherms fitted Langmuir model quite well (*R*^2^ > 0.999) (Fig. 7a and b). The Hg(ii) removal capacity of S@TP-EDDA COF was measured to be 718 mg g^−1^ (pH = 6), which is higher than those of many porous materials, like COF-LZU8 (236 mg g^−1^),^35^ MOF Zr-DMBD (197 mg g^−1^),^48^ activated carbon (518 mg g^−1^),^49^ and porous silica (600 mg g^−1^).^50^ This good adsorption capacity of TP-EDDA COF can be attributed to the 1D open channels, the highly dispersed sulfur and enough S^2−^ bonding sites within the TP-EDDA COF. In contract, no Hg(ii) was observed for TP-EDDA COF, suggesting that the adsorption of Hg(ii) was attributed to the sulfur within the COF. The removal effectiveness of the COF is also an important factor for a Hg(ii) adsorbent. The adsorption kinetics was investigated for a system with HgCl_2_ (50 mL; 10 ppm, pH = 6) and TP-EDDA COF (25 mg) at 25 °C. A quick Hg(ii) removal was observed, as reflected by the fact that over 99% of Hg(ii) was removed within 10 min. The removal process can be finished within *ca.* 20 min and the initial Hg(ii) concentration was decreased from 10 ppm to 0.01 ppm (Fig. 7c). The adsorption rate constant, *k*_2_, was calculated to be 3.02 g mg^−1^ min^−1^ (Fig. 7d and e) by fitting with a pseudo-second-order model. Such a high adsorption rate for Hg(ii) can also be attributed to the 1D open channels of the TP-EDDA COF and the widely dispersed sulfur atoms throughout the skeleton within the TP-EDDA COF.

**Fig. 8:**
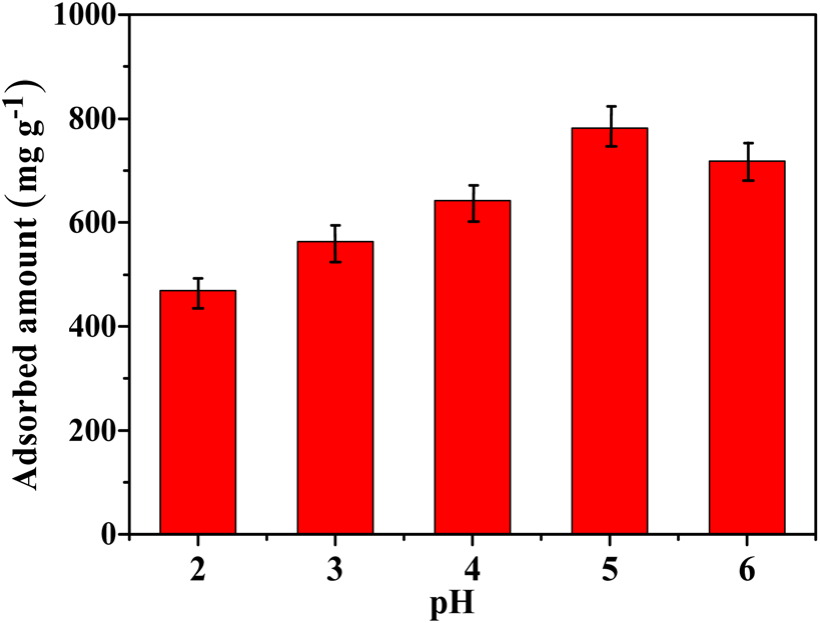
The effect of pH on the Hg(ii) adsorption capability of TP-EDDA COF.

It is known that the pH of a solution plays an important role in the adsorption process. Therefore, the effect of pH on Hg(ii) adsorption was further investigated in this work. Given the fact that Hg(OH)_2_ precipitate would form when the pH of solution is higher than 6 and the TP-EDDA COF will be destroyed when the pH is less than 1. Thus, the Hg(ii) adsorption behavior was investigated at pH ranging from 2 to 6. As shown in Fig. 8, the optimal pH for Hg(ii) adsorption is 5. At lower pH values, S^2−^ can be neutralized by H^+^ that will decrease the adsorption capability of Hg(ii). When the pH is higher, Hg(ii) will form Hg(OH)_2_ precipitate due to the lower *K*_sp_ of Hg(OH)_2_, causing a decreased adsorption capability. Besides, it is of great importance for evaluating its reusability performance of TP-EDDA COF adsorbent. It is evident (Fig. S7†) that the adsorption capability of S@TP-EDDA COF decreased gradually, but still remained about 80% adsorption performance (564 mg g^−1^) after 5 cycles, suggesting the good reusability of the COF. The decrease in adsorption capability may be due to the lost sulfur, blocked pores or decreased crystallization of the COF after the cyclic treatments.

## 4 Conclusions

Covalent organic frameworks (COFs) have been promising candidates for removing heavy metal Hg(ii) ions; however, it is still a difficulty to design and construct a crystalline functional COF through either a “bottom-up” or post-synthetic synthesis strategy. In this work, we prepared an alkynyl (–C≡C–) based COF, TP-EDDA COF, as an adsorbent for Hg(ii) removal. After loading sulfur vapor into the pores of the COF, charge transfer happened from the π-conjugated structure of the COF to sulfur atoms, which gave S^2−^ to bond Hg(ii) effectively. A good Hg(ii) removal capacity and high removal effectiveness for the COF were achieved, which can be attributed to the 1dimensional (1D) open channel structure, highly dispersed sulfur atoms and enough sulfur interaction sites with Hg(ii) throughout the skeleton of the COF. This strategy may open up a simple and effective way for removing toxic metals and suggests the promising potential of COFs to cope with various pollution issues.

## Author contributions

Conceptualization, S. W., Y. X., X. S. and Y. G.; methodology, S. W., J. W., Q. Y., and J. Q.; software, H. H.; validation, H. H. and Y. G.; formal analysis, S. W.; investigation, Y. X., X. S. and S. X.; resources, X. S., S. X., H. H. and Y. G.; writing – original draft preparation, S. W. and Y. X.; writing – review and editing, Y. G.; supervision, S. X. and Y. G. All authors have read and agreed to the published version of the manuscript.

## Conflicts of interest

There are no conflicts to declare.

## Supplementary Material

RA-012-D2RA06838A-s001
