# Supplementary material for: Adsorption of sulfur into an alkynyl-based covalent organic framework for mercury removal
Source: RSC Adv. 2022 Dec 12;12(54):35445–51. doi: 10.1039/d2ra06838a (PMC9742859; doi:10.1039/d2ra06838a)
Supplement: RA-012-D2RA06838A-s001 [file RA-012-D2RA06838A-s001.pdf]

Electronic Supplementary Material (ESI) for RSC Advances.
This journal is © The Royal Society of Chemistry 2022

# Supplementary Information

## Adsorption of Sulfur into an Alkynyl-based Covalent Organic Framework for Mercury Removal

Shenglin Wang,[†a] Yingxiang Xin,[†b] Hui Hu,[a] Xiaofang Su,[*a] Jifeng Wu,[a] Qianqian Yan,[a] Jiaying Qian,[a] Songtao Xiao[*c] and Yanan Gao[*a]

### Synthesis of 4,4′-(ethyne-1,2-diyl)dianiline (EDDA)

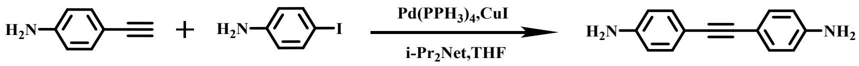


4,4′-(Ethyne-1,2-diyl)dianiline (EDDA) was synthesized according to a modified method.[S1] Typically, to a 50 mL double-necked round-bottomed flask was added $PdCl_2(PPh_3)_2$ (0.18g, 0.256 mmol), CuI (0.0488 g, 0.256 mmol), *p*-ethynylaniline (1.12 g, 5.12 mmol) and *p*-iodoaniline (0.6 g, 5.12 mmol). The flask was degassed and charged with dried $N_2$ by three freeze-pump-thaw cycles and then loaded with dried THF (15 mL) and *N*-ethyldiisopropylamine (2 mL). The mixture was stirred for 24 h at room temperature and the precipitate was separated by filtration. The solid was washed with $CH_2Cl_2$ until the washing liquid was colourless. The organic phase was collected and washed with saturated NaCl solution and dried with anhydrous $MgSO_4$. The solvent was removed by rotary evaporation. The crude product was purified by column chromatography on $Al_2O_3$ (200-300 meshes) eluted with (petroleum ether/$CH_2Cl_2$ 2:1) to give pure pale brown EDDA product (260 mg) in 24.3% yield.

$^1$H NMR ($CDCl_3$, 400 MHz) δ = 7.31 (d,4H, -Ar-H), δ = 6.63 (d,4H, Ar-H), δ = 3.7 (s,4H, -$NH_2$). $^1$H NMR spectrum was shown in Fig S1.

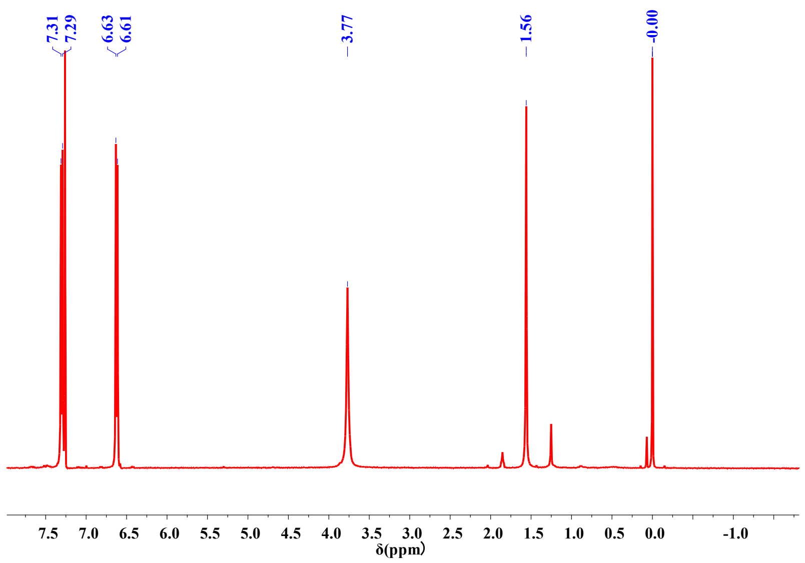


**Fig S1.** $^1$H NMR spectra of EDDA.

### Synthesis of 1,3,5-triformylphloroglucinol (TP)

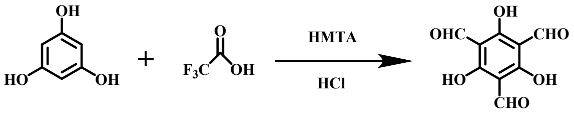


1,3,5-Triformylphloroglucinol (TP) was synthesized according to a modified method.[S2] Typically, to a 50 mL three-necked round-bottomed flask was added hexamethylene tetramine (14 g, 100 mmol), phloroglucinol (6.3 g, 50 mmol), and 100 mL of trifluoroacetic acid. The reaction was heated at 100 °C for 3 hours under $N_2$ production and 150 mL of hydrochloric acid was added. The reaction was carried out for another one hour and cooled down to room temperature. Celite was added into the reaction and the mixture was stirred for 30 minutes. The precipitate was separated by filtration and washed with $CHCl_3$. The organic phase was collected and dried with anhydrous $MgSO_4$. The solvent was removed by rotary evaporation and the white solid product (1.4 g) was obtained after drying under vacuum at 80 °C for 12 hours. The yield was calculated to be 14%.

$^1$H NMR ($CDCl_3$, 400 MHz), δ = 14.12 (s,3H, -OH), δ = 10.16 (s,3H, -CHO). The $^1$H NMR spectrum was shown in Fig S2.

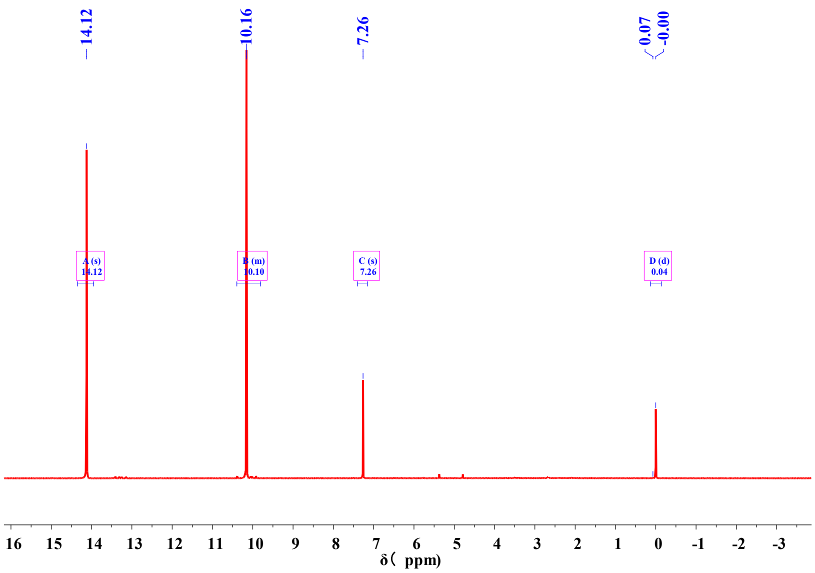


**Fig S2.** $^1$H NMR spectra of TP.

**Synthesis of TP-EDDA COF**

The optimum reaction condition for the formation of crystalline TP-EDDA COF was screened. TP (12.6 mg, 0.06 mmol) and EDDA (19.8 mg, 0.09 mmol) were loaded in a glass ampule vessel (10 mL). Then, a 1.0 mL mixed solution (for instance, mesitylene/1,4-dioxane, 1/1 by volume) was added to the vessel. After the mixture was sonicated for 10 min, 0.1 mL acetic acid (6.0 M) was rapidly added. The vessel was sonicated for another 5 min and then flash-frozen in liquid nitrogen. The reaction system was degassed through freeze-pump-thaw cycles for three times. The system was sealed (< 4 Pa) with a flame, and then heated at 120 °C for 3 days. A precipitate was obtained after filtration and washed thoroughly with N,N-dimethylformamide (DMF) and tetrahydrofuran (THF), successively, to produce an orange powder. The powder was dried at 120 °C under vacuum overnight to give the final product. The mixed solvent (1.0 mL) used for the synthesis of TP-EDDA COF included (1) dioxane/mesitylene (1:1, v/v); (2) dioxane; (3) 1,2-dichlorobenzene (o-DCB)/n-butanol (BuOH) (1:1, v/v) and (4) dioxane/mesitylene (3:2, v/v). The PXRD patters of obtained TP-EDDA COF were shown in Fig S3. It is evident that TP-EDDA COF can be obtained under several different reaction conditions. The optimum reaction condition, i.e., mesitylene/1,4-dioxane, 1/1 by volume was discussed in detail in the main text of the paper. The $S_{BET}$ and yield of TP-EDDA COF obtained under different reaction conditions were listed in Table S1.

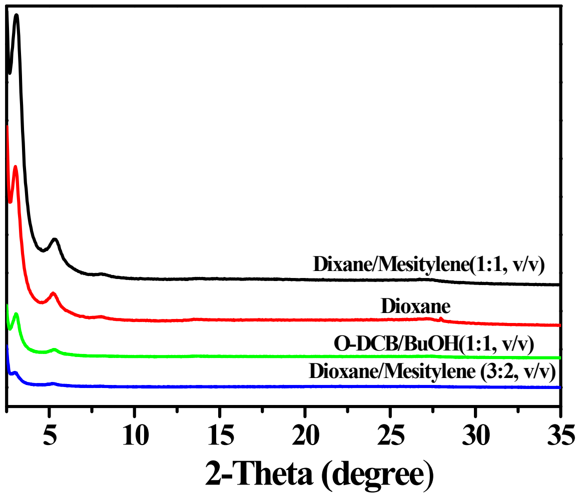


**Fig S3.** PXRD patterns of TP-EDDA COF synthesized in different solvent systems.

**Table 1.** Surface area and yield of TP-EDDA COF obtained in different solvents.

| Solvents | Dioxane/Mesitylene (1:1) | Dioxane | o-DCB/BuOH (1:1) | Dioxane/Mesitylene (3:2) |
|---|---|---|---|---|
| $S_{BET}$ ($m^2/g$) | 1183 | 780 | 236 | 301 |
| Yield (%) | 73.8 | 75.1 | 70.5 | 80 |

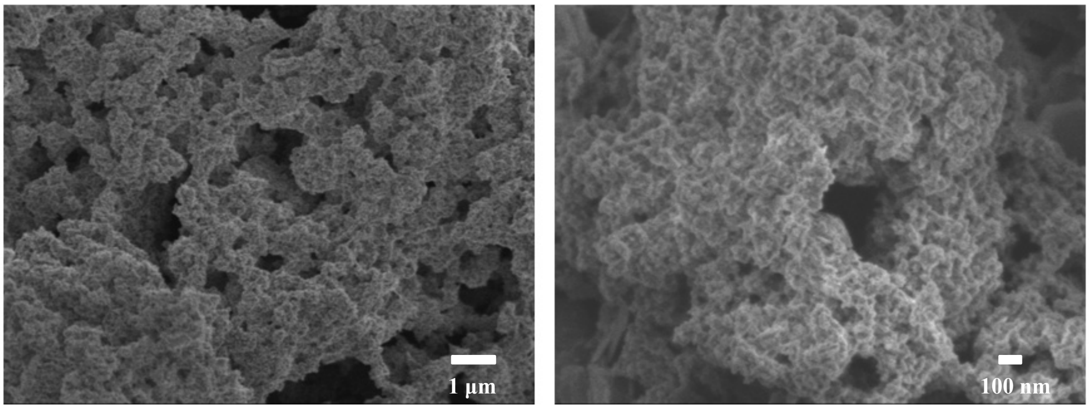


**Fig S4.** SEM images of TP-EDDA COF at different magnifications.

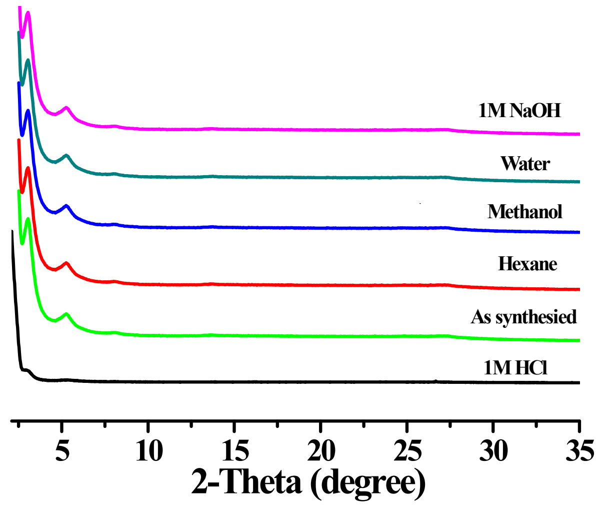


**Fig S5.** PXRD pattern of TP-EDDA COF after treatment with various harsh conditions.

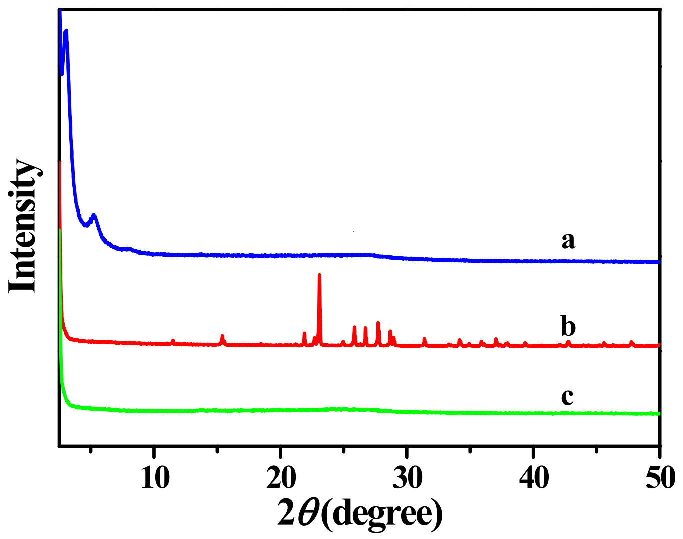


**Fig S6.** PXRD patterns of TP-EDDA COF (a), sulfur (b) and S@TP-EDDA COF (c).

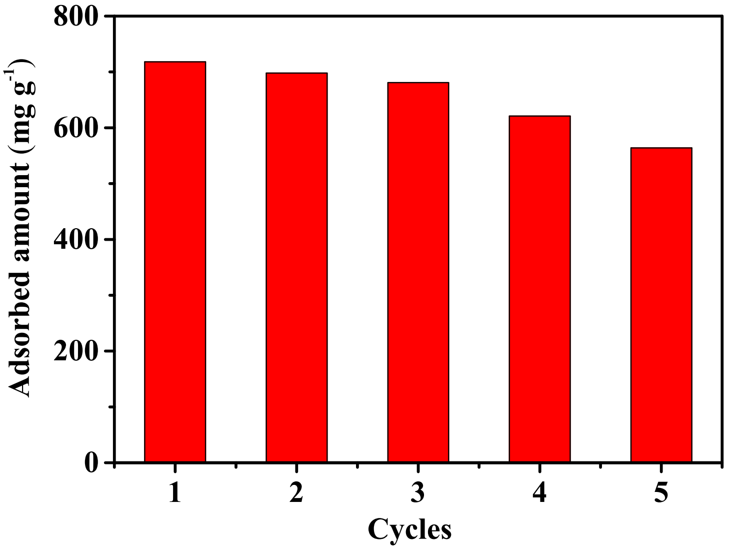


**Fig S7.** Recyclability of the S@TP-EDDA COF adsorbent (pH =6).

**References**

[S1] D. Nishimura, T. Oshikiri, Y. Takashima, A. Hashidzume, H. Yamaguchi, A. Harada, *J. Org. Chem*. **2008**, *73*, 2496-2502.

[S2] J. H. Chong, M. Sauer, B. O. Patrick, M. J. MacLachlan, *Org. Lett*. **2003**, *5*, 3823-3826.
